# Aggressive Disease Course of Multiple Myeloma with Concomitant ALK-Negative Anaplastic Large Cell Lymphoma: A Case Report with an Unusual Presentation

**DOI:** 10.1155/2020/6309736

**Published:** 2020-01-16

**Authors:** Michela Staderini, Lara Mannelli, Elisabetta Antonioli, Benedetta Puccini, Valentina Berti, Francesco Mungai, Federica Vergoni, Valentina Carrai, Luigi Rigacci, Alberto Bosi

**Affiliations:** ^1^Haematology Unit, Careggi University Hospital, Florence, Italy; ^2^Medical Genetics, University of Siena, Siena, Italy; ^3^Nuclear Medicine Unit, Department of Biomedical Experimental and Clinical Sciences, Careggi University Hospital, Florence, Italy; ^4^Diagnostic Radiology Unit, Careggi University Hospital, Florence, Italy; ^5^Pathological Anatomy Unit, Careggi University Hospital, Florence, Italy; ^6^Hematology and Stem Cell Transplant Unit, San Camillo Forlanini Hospital, Rome, Italy

## Abstract

ALK-negative anaplastic large cell lymphoma is a rare T-cell neoplasm with an aggressive course requiring prompt diagnostic work-up and treatment. Few cases of concomitant multiple myeloma and T-cell neoplasm are described in the literature, mainly regarding primary cutaneous anaplastic large cell lymphoma. We present the case of a 65-year-old man, simultaneously diagnosed with ALK-negative anaplastic large cell lymphoma with extranodal localization in the gastrocnemius muscle (stage 1AE) and IgG lambda multiple myeloma (ISS 2, Durie-Salmon stage 3A). Both diseases required therapeutic intervention due to the high proliferative index of lymphoma and the presence of bone lesions attributable to myeloma. The therapeutic program initially included chemotherapy (cyclophosphamide, doxorubicin, vincristine, etoposide, and prednisone; CHOEP), radiotherapy on the leg, bortezomib, and then consolidation with autologous hematopoietic stem cell transplantation. Despite being on bortezomib treatment and waiting for transplantation, the patient experienced an early myeloma progression that turned out to be refractory to second-line lenalidomide-based treatment. To our knowledge, this is the first case of concurrent diagnosis of extranodal ALK-negative anaplastic large cell lymphoma of the muscle and multiple myeloma. Simultaneous onset can be challenging for clinicians as both diseases may have an aggressive course requiring multiple treatments with increased risk of toxicity and complicated management.

## 1. Introduction

ALK-negative anaplastic large cell lymphoma (ALK-ALCL) is a rare CD30+ T-cell neoplasm involving both lymph nodes and extranodal sites, often presenting with B symptoms and advanced stage disease [1]. Primary involvement of the skeletal muscles is extremely rare in adults and has been associated with rapid dissemination and poor prognosis [2–4].

On the other side, multiple myeloma (MM) is characterized by clonal plasma cell (PC) infiltration in the bone marrow and, rarely, in extramedullary sites, leading to signs of organ damage typically summarized by the acronym CRAB (hypercalcemia, renal failure, anaemia, and bone lesions).

Few cases of concomitant diagnosis of multiple myeloma and T-cell neoplasms are described, mainly regarding CD30+ cutaneous anaplastic large cell lymphoma. Often the plasma cell dyscrasia precedes the diagnosis of the T-cell neoplasm [5–7]. Some authors have hypothesized a causal relationship between the two diseases, but the potential pathogenic mechanism has not been fully understood [7]. Currently, only one case of contemporary diagnosis of aggressive ALK-negative ALCL and IgA lambda multiple myeloma is described in the literature [8]; that case was characterized by 15% plasma cell infiltration in the bone marrow (suggestive of smoldering MM), and no information on follow-up or response to treatment was available because the patient died quickly after diagnosis.

To the best of our knowledge, this is the first report of a simultaneous diagnosis of symptomatic extranodal ALK− ALCL and MM, requiring to start a treatment aiming to target both diseases.

## 2. Case Presentation

In June 2017, a Caucasian 65-year-old man was admitted to the Hematology Unit of Careggi Hospital (Florence, Italy) with swelling and pain in his right leg. At MRI, a large spindle-shaped lesion (size 3 × 2.2 × 8.4 cm) was detected at his right gastrocnemius muscle. The lesion had the same signal intensity of the surrounding normal muscular tissue in the T1-weighted sequence and intermediate signal intensity in the T2-weighted sequence; it was hyperintense in sequences with fat suppression and showed a high contrast-enhancement.

**Figure 1:**
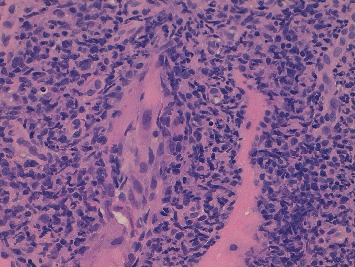
Core needle biopsy of the lesion in the right gastrocnemius muscle. H&E stain showing a dense infiltrate of large pleomorphic cells with prominent nucleoli and horseshoe or kidney-shaped nuclei (hallmark cells) consistent with ALCL.

**Figure 2:**
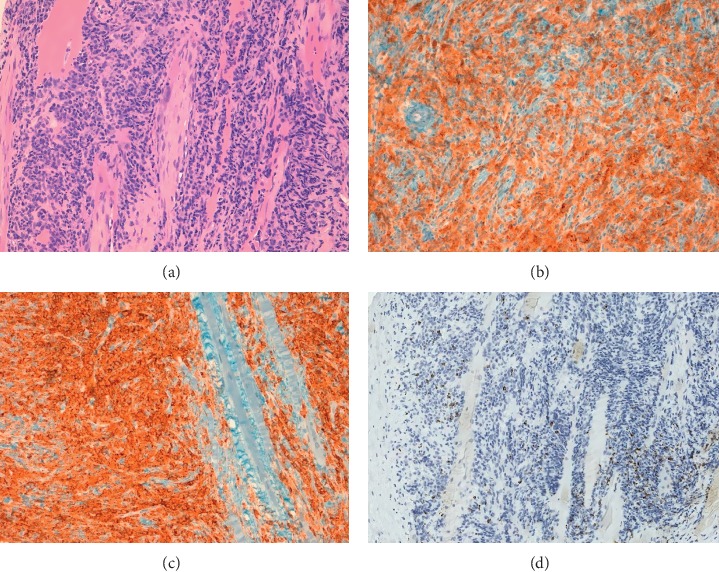
Immunohistochemical features of the lymphoma. (a) Hematoxylin and eosin staining; (b) CD30 and (c) CD4 immunostaining showing a strong and uniform expression in the neoplastic cells; (d) TIA1 immunostaining; this marker was only expressed in a minority of cells (−/+).

He underwent a core needle biopsy of the lesion with morphologic examination demonstrating a dense infiltrate of large pleomorphic cells with prominent nucleoli, many of which having horseshoe or kidney-shaped eccentric nuclei resembling hallmark cells (Figure 1). Immunohistochemical staining showed expression of CD45 and CD4, strong and uniform expression of CD30, and negativity of CD19, CD20, Pax-5, CD3, CD5, CD8, EMA, Perforin, CD56, CD34, and ALK in the neoplastic cells; TIA1 was only expressed in a minority of cells (−/+); MIB-1 was >90%. These findings were consistent with ALK-negative anaplastic large cell lymphoma (Figure 2).

The patient did not complain about any systemic symptom; his physical exam was normal. He had no previous medical history of cancer or autoimmune diseases. In his medical records, atrial fibrillation and ventricular flutter on treatment with beta-blockers were reported. Blood tests showed normal white blood cell and platelet counts, with mild anaemia (haemoglobin 12.5 g/d); LDH and liver enzymes were normal, creatinine 1.25 mg/dl, calculated GFR 54 ml/min, and beta2 microglobulin 4.5 mg/L. In addition, a serum IgG lambda monoclonal component (1.53 g/dl) was found, with increased lambda light chains (106 mg/L), and the sFLC ratio was lower than 100, albumin 33.7 g/L, and calcium 8.9 mg/dl.

**Figure 3:**
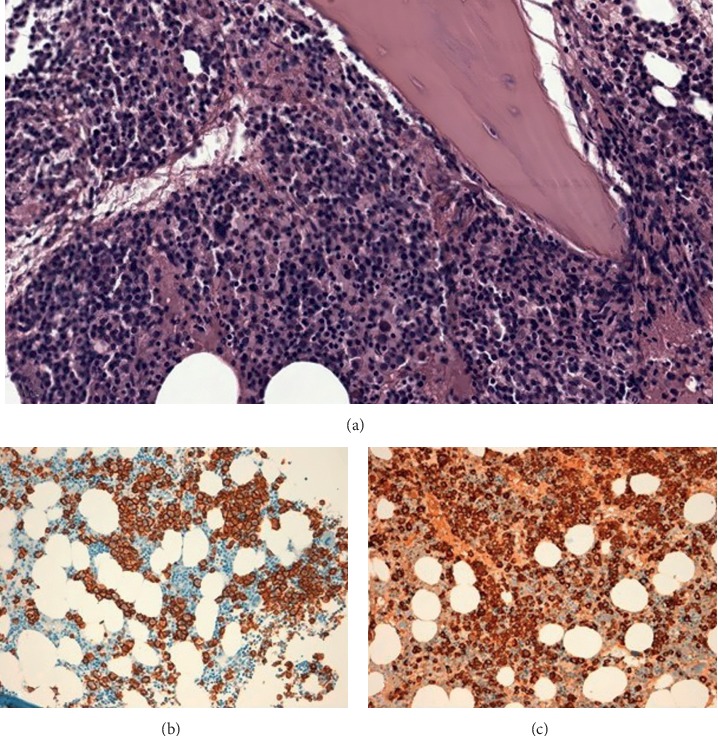
Bone marrow biopsy performed at diagnosis showed plasma cell infiltration with lambda-chain restriction. (a) H&E staining; (b) CD138 staining; (c) lambda staining.

A whole-body computed tomography (CT) scan did not show any other pathologic finding; in particular, no bone lytic lesions were revealed. In order to complete lymphoma staging procedures, a bone marrow biopsy was performed, and a relevant infiltration (roughly 70%) by atypical plasma cells with lambda light-chain restriction was detected (Figure 3).

Multiparameter flow cytometry on the bone marrow sample showed plasma cells, accounting for 45% of cellularity, that were positive for CD138 and CD38 and negative for CD19, CD56, and CD45; CD117 was partially expressed. No signs of bone marrow clonal T-cell infiltration were found. Cytogenetic analysis was not performed because of insufficient bone marrow sample.

**Figure 4:**
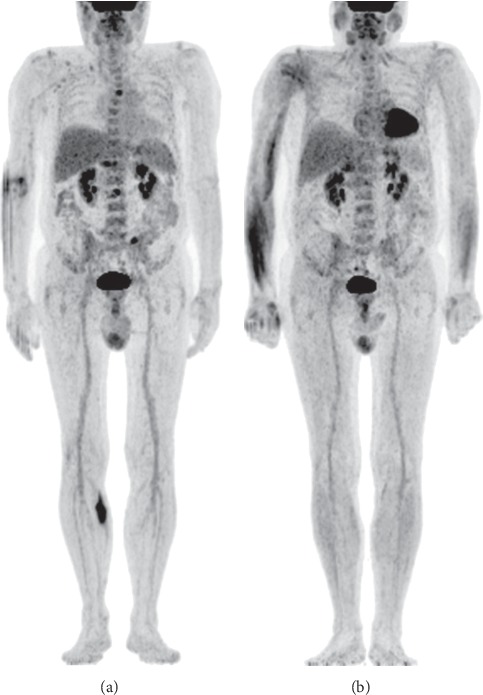
F18-FDG PET/CT performed at staging showed an intensely hypermetabolic lesion in the right leg and revealed three additional vertebral hypermetabolic lesions in D5, L2, and S1 (a). After chemotherapy, the hypermetabolic lesions in the right leg, D5, and L2 had completely disappeared, with persistence of moderate metabolic activity in the left sacral lesion (DS = 3) (b).

An F18-FDG PET/CT confirmed the lesion in the right gastrocnemius muscle, which was intensely hypermetabolic, and revealed three additional vertebral hypermetabolic lesions in D5, L2, and S1, that were ascribed to myeloma (Figure 4(a)).

Based on these findings, the patient was diagnosed with an aggressive CD30+ ALK-negative ALCL (stage 1AE) and a concomitant IgG lambda multiple myeloma (MM, ISS score 2, and Durie-Salmon stage IIIA). Due to one MM-defining event (bone lesions at F18-FDG PET/CT) and one biomarker of malignancy (bone marrow infiltration over 60%), myeloma was defined as symptomatic, thus indicating the need to start an effective treatment.

The concurrence of two aggressive diseases led us to adopt a treatment approach taking into account the high proliferative index of lymphoma and therefore was based on the immediate application of standard ALCL treatment, followed by a consolidation program including autologous stem cell transplantation (ASCT), preceded by a conditioning regimen also active on myeloma.

From August 2017 to November 2017, the patient received 4 courses of polychemotherapy according to the CHOEP regimen (21-day cycles of cyclophosphamide 750 mg/sqm, doxorubicin 50 mg/sqm, and vincristine 1.4 mg/sqm on day 1; etoposide 100 mg/sqm on days 1–3; prednisone 100 mg/sqm on days 1–5).

In January 2018, after chemotherapy, an MRI showed a complete response of the ALCL lesion, and a 18F-FDG PET/CT revealed the complete disappearance of the right gastrocnemius muscle and of D5 and L2 lesions, with persistence of moderate metabolic activity in the left sacral lesion (Deauville score = 3) (Figure 4(b)). Serum M-component decreased (0.4 gr/dl), and a re-evaluation of bone marrow biopsy displayed the reduction of plasma cell infiltration to 10% of marrow cellularity. As such, MM was in partial response. On February 2018, peripheral blood stem cell (PBSC) mobilization was achieved following high-dose cyclophosphamide (2 gr/sqm) and granulocyte-colony stimulating factor (G-CSF), and an amount of CD34+ cells adequate for a single ASCT was collected. Then, in March 2018, the patient received localized radiotherapy on the right gastrocnemius muscle for a total of 36 Gy, without any relevant complication. Waiting to be transplanted, on April 2018, the patient started VD treatment (bortezomib 1.3 mg/smq twice a week, days 1–4–8–11 and oral dexamethasone 40 mg, days 1-2–4-5–8-9–11-12, cycles every 21 days), completing two cycles. On June 2018, during the third course, he was hospitalized because of an *E. coli* infection complicated by sepsis and congestive heart failure; he was discharged one month later. Serum M-protein remained permanently around 0.3 g/dl, and serum lambda light chains were 30 mg/L, confirming a MM partial response.

Unfortunately, on July 2018, few days later, he was hospitalized again due to paraplegia and neurogenic bladder dysfunction. An MRI of the spine showed a spinal cord compression due to neoplastic tissue extending from vertebrae D6 to D10 (size 10 × 5 × 2.5 cm). Considering the severity of the neurologic impairment, a new biopsy of the lesion was not performed, and radiotherapy in association to high-dose dexamethasone was immediately started, obtaining a rapid neurologic improvement.

M-protein kept stable (0.3 g/dl), but lambda sFLC were increased to 860 mg/L. A new bone marrow biopsy showed an increased monoclonal plasma cell infiltration (50% of marrow cellularity) with morphologic atypia and aberrant phenotype. Fluorescent in situ hybridization (FISH) performed on purified bone marrow plasma cells, which was not available at the diagnosis, showed *t*(14;16) in 100% and del17p13 in 64% of the cells analyzed. On August 2018, we started a second line therapy with weekly daratumumab in combination with lenalidomide and dexamethasone (DRd). After one cycle, the patient's conditions deteriorated, and a new MRI of the spine revealed two additional neoplastic lesions that extended from vertebrae D2 to D6 and from vertebrae D12 to L4. In September 2018, the patient was deceased due to disease progression.

## 3. Discussion

ALK− ALCL usually presents with advanced stage disease (stage III-IV), B symptoms, lymph nodes, and extranodal tissue involvement [1]. The median age at presentation of ALK− ALCL patients is 54–61 years [9].

Our case instead presented with an extranodal noncutaneous ALK− ALCL showing muscular involvement as a unique lesion without systemic symptoms. This clinical onset coincided with a symptomatic IgG lambda MM. According to the last edition of WHO classification, ALK− ALCL is defined as a CD30+ T-cell neoplasm that is not reproducibly distinguishable by morphology from ALK-positive (ALK+) ALCL, but lacks ALK protein expression [1]. T-cell/cytotoxic markers can be lacking in ALCL as well as EMA which is only positive in 43% of the cases [10]. While both Pax-5 negativity and cell morphology helped us to rule out classical Hodgkin lymphoma, distinguishing ALCL from PTCL-NOS can be challenging. The occurrence of hallmark cells together with strong and uniform CD30 expression in the neoplastic proliferation appeared more consistent with ALCL than PTCL-NOS in this case.

Regarding concomitant diagnosis of T-cell lymphoma and MM, in the few other cases described in the literature [5–7], the diagnosis of a T-cell neoplasm occurred later than the diagnosis of MM, leading to surmise a pathogenic role for acquired cellular immunity impairment, on turn caused by PC disorders or previous therapies. In our patient the relationship between the two neoplasms was unclear, and the chronologic sequence of onset could not be proven. Nevertheless, the patient did not have any prior history of MGUS or other plasma cell dyscrasias. Moreover, the incidence of lymphoproliferative disorders is generally increased in immunosuppressed patients, but our patient was not previously treated with any chemo-radiotherapy and blood tests excluded an active viral infection.

The optimal treatment for ALK-negative ALCL is still debated because of the rarity of the disease and the related lack of prospective randomized trials. ALK− ALCL generally responds well to doxorubicin-containing chemotherapy, but frequently relapses [11]. The role of autologous stem cell transplant in first remission in ALCL has been investigated in small trials with 5-year overall survival (OS) rates of up to 80%. However, in many of these studies, ALK expression was not assessed [12, 13]. The prognosis of ALK− ALCL patients is poor, with a 5-year (OS) of 30–49%, versus 70–86% in ALK+ ALCL [14, 15]. On the contrary, multiple myeloma treatment is well standardized and includes upfront autologous stem cell transplantation for younger patients [16]. However, the prognosis can be quite heterogeneous according to cytogenetic and biochemical markers [17]: specifically, the outcome of patients harbouring one of the high-risk chromosomal aberrations, such as *t*(4;14), *t*(14;16), *t*(14;20), and especially del17p, is dismal and not completely overcome by the new drug combinations so far [18].

In our patient, both diseases required therapeutic intervention. Because of the high proliferation index (MIB-1 > 90%), we decided to treat lymphoma first. Afterwards, we observed an aggressive behaviour and a rapid progression of multiple myeloma which led the patient to death, eventually explained by the double high-risk cytogenetic alterations identified at relapse.

Unfortunately, no cytogenetic data were available at diagnosis, and we cannot exclude a clonal selection secondary to chemotherapy treatment for lymphoma.
